# Understanding the Rising Incidence of Early-Onset Colorectal Cancer: Life-Course Determinants and Opportunities for Primary Prevention

**DOI:** 10.3390/cancers18172852

**Published:** 2026-09-03

**Authors:** Antoni Alegre-Martinez, Luis Cabañas-Alite, Ines E. Fernández-Benet, Manuel Sánchez-Casalongue, Jose M. Martin-Moreno

**Affiliations:** 1 Biomedical Research Institute—INCLIVA, University of Valencia, 46010 Valencia, Spain; aalegre@incliva.es (A.A.-M.); lcabanas@incliva.es (L.C.-A.); ifernandez@incliva.es (I.E.F.-B.); masanchez@incliva.es (M.S.-C.); 2 Faculty of Health Sciences, Miguel de Cervantes European University, C. del Padre Julio Chevalier, 2, 47012 Valladolid, Spain; 3 Department of Preventive Medicine, Medical School, University of Valencia, 46010 Valencia, Spain

**Keywords:** early-onset colorectal cancer, young-onset colorectal cancer, modifiable risk factors, primary prevention, life-course epidemiology, obesity, metabolic syndrome, diet, sedentary behavior, inflammatory bowel disease

## Abstract

**Background/Objectives**: Early-onset colorectal cancer (EOCRC), diagnosed before age 50 years, is increasing in many countries, but its causes remain uncertain. Evidence on potentially modifiable determinants is heterogeneous and vulnerable to retrospective assessment and residual confounding. We synthesized metabolic, behavioral, dietary, medication-related, clinical, and socioeconomic factors, emphasizing consistency and life-course relevance. **Methods**: Two core PubMed searches covering publications from 1 January 2010 through 1 June 2026 were supplemented by targeted PubMed searches and reference-list screening. The present review cited 75 journal publications and synthesized the findings qualitatively; no formal risk-of-bias assessment, certainty-of-evidence grading, or de novo quantitative pooling was performed. **Results**: Evidence was most consistent for excess adiposity and cardiometabolic dysfunction, with earlier and persistent exposure and cardiometabolic clustering potentially more relevant. Inflammatory bowel disease emerged as a clinically distinct high-risk condition. Sedentary behavior, smoking, and higher alcohol consumption showed recurrent but heterogeneous associations. Dietary findings varied by pattern and component: sugar-sweetened beverages and lower-quality dietary patterns showed positive associations, whereas fiber- and plant-rich diets, calcium, folate, and vitamin D showed possible inverse associations. Dietary evidence was largely retrospective and does not establish causality. Evidence for aspirin and antibiotics was limited and vulnerable to confounding, precluding causal inference. Most patterns resembled later-onset CRC. **Conclusions**: Current evidence does not support a wholly distinct set of environmental determinants for EOCRC. Earlier exposure, longer duration, and cumulative interaction of CRC risk factors are plausible explanations but remain incompletely tested. Evidence supports life-course primary prevention, but no single factor should determine screening eligibility in isolation. Prospective studies with repeated exposure measurement are needed.

## Simple Summary

Colorectal cancer diagnosed before age 50 is becoming more common in many countries, although the reasons remain uncertain. This review examined 75 publications on lifestyle, metabolic, clinical, medication-related, and social factors associated with early-onset disease. The clearest associations involved excess body weight and poor metabolic health. Physical inactivity, smoking, alcohol consumption, and dietary patterns showed less consistent associations, while inflammatory bowel disease emerged as a clinical risk condition. Evidence on medications and social conditions was limited. Many of these factors influence colorectal cancer at older ages; their relevance to earlier-onset disease may depend on when exposure begins, its duration, and how exposures interact over time. The findings support prevention throughout the life course with healthier food and activity environments, tobacco control, reduced harmful alcohol use, and better metabolic health. However, observational evidence does not establish that any single factor causes early-onset colorectal cancer or should independently determine screening eligibility.

## 1. Introduction

Colorectal cancer epidemiology shows a marked age divergence: incidence has stabilized or declined among adults ≥50 years in settings with established screening, whereas early-onset colorectal cancer (EOCRC) has risen across multiple countries, commonly by ~1–3% annually [1,2,3,4,5,6]. International comparisons across 42 countries reinforce this divergence, with CRC incidence increasing among adults aged 20–49 years in 88% of countries and trends being more unfavorable in younger than older adults in 69% [7]. EOCRC also forms part of a broader increase in cancers among young adults, exposing prevention and care pathways still largely designed around older populations [8]. Despite large relative increases, EOCRC remains less common in absolute terms than later-onset colorectal cancer, an important consideration when interpreting its growing population burden [9]. A consistent birth-cohort signal suggests that risk is increasing more steeply in recent generations, supporting etiologic models centered on early-life, life-course, socioeconomic, and lifestyle transitions rather than exclusively adult-onset exposures [1,10,11,12,13,14].

These generational patterns make it particularly relevant to examine potentially modifiable exposures across the life course, alongside clinical risk conditions and socioeconomic context. Adiposity, metabolic health, diet, physical activity, tobacco and alcohol use, and medication exposure may accumulate from childhood through adulthood. At the same time, inflammatory conditions and socioeconomic context may further shape risk and opportunities for prevention. Early-life exposomic and microbiome changes, together with potential age-related molecular and immune differences, may also influence disease expression before midlife [15]. Emerging cohort and proteomic evidence further links accelerated systemic and adipose-tissue biological aging with early-onset gastrointestinal and colorectal cancer, although these biomarkers remain investigational [16]. However, the observed increase cannot be attributed to any single exposure, and the available literature remains predominantly observational, heterogeneous in exposure measurement, timing, confounder adjustment, and outcome definition. These associations should therefore not be interpreted as proof of causality, but as signals that may inform prevention hypotheses and priorities for prospective research [1,9,10,11,12,13].

This narrative review does not seek to assign the rising EOCRC burden to a single explanatory mechanism or to catalog every proposed environmental exposure. Instead, it synthesizes evidence on metabolic, behavioral, dietary, medication-related, clinical, and socioeconomic determinants. Priority is given to consistency across studies, pooled estimates where available, exposure timing and persistence, and the relevance of findings for primary prevention. Particular attention is given to potential threats to causal inference, including reverse causation, confounding by indication, healthcare utilization bias, and imprecise exposure assessment. The aim is not to define screening eligibility based on isolated associations, but to clarify prevention priorities, clinically relevant high-risk contexts, and gaps that require prospective life-course research.

### Objectives

1. To synthesize evidence on metabolic, behavioral, dietary, medication-related, clinical, and socioeconomic determinants associated with EOCRC.
2. To assess the consistency and life-course relevance of these associations, including exposure timing, persistence, and dose–response patterns.
3. To identify implications for primary prevention, clinical vigilance, and future life-course research.

## 2. Materials and Methods

### 2.1. Study Design and Scope

This narrative review examined potentially modifiable exposures, clinical risk conditions, and socioeconomic or contextual determinants associated with EOCRC. It was developed within the ONCODIR Work Package 2 framework (HORIZON-MISS-2022-CANCER-01) and forms one of two companion narrative reviews derived from a shared literature-identification process. The present review was restricted to domains relevant to primary prevention and life-course exposure. The review domains included adiposity and weight trajectories; physical activity and sedentary behavior; cardiometabolic dysfunction; inflammatory bowel disease and selected comorbidities; medication and antibiotic exposure; tobacco and alcohol use; dietary patterns and components; and socioeconomic context. Tumor location, screening, prediagnostic symptoms, demographic markers, family history, and inherited susceptibility were addressed separately in the companion review, “Closing the Diagnostic Gap in Early-Onset Colorectal Cancer: Red-Flag Symptoms, Screening, and Inherited Risk” (Martin-Moreno et al., 2026) [17].

### 2.2. Literature Search

Two complementary core PubMed searches were performed to identify meta-analyses and primary observational studies, including cohort, case–control, prospective, and population-based designs. The first search targeted published meta-analyses to identify pooled evidence, whereas the second targeted primary observational studies to capture individual-study evidence not represented in meta-analyses. Publications dated from 1 January 2010 through 1 June 2026 were considered in the core searches.

To identify meta-analyses, we used the following PubMed strategy: (“colorectal cancer”[Title/Abstract] OR “colon cancer”[Title/Abstract] OR “rectal cancer”[Title/Abstract]) AND (“early-onset”[Title/Abstract] OR “young adults”[Title/Abstract] OR “under 50”[Title/Abstract] OR “younger than 50”[Title/Abstract]) AND (“risk factor”[Title/Abstract] OR “risk factors”[Title/Abstract] OR “etiology”[Title/Abstract] OR “determinant”[Title/Abstract] OR “associated with”[Title/Abstract]) AND (Meta-Analysis[ptyp]) AND (“1 January 2010“[Date-Publication]: “1 June 2026”[Date-Publication]).

To identify primary observational studies, we used: (“colorectal cancer”[Title/Abstract] OR “colon cancer”[Title/Abstract] OR “rectal cancer”[Title/Abstract]) AND (“early-onset”[Title/Abstract] OR “young adults”[Title/Abstract] OR “under 50”[Title/Abstract] OR “younger than 50”[Title/Abstract]) AND (“risk factor”[Title/Abstract] OR “risk factors”[Title/Abstract] OR “etiology”[Title/Abstract] OR “associated with”[Title/Abstract]) AND (cohort[Title/Abstract] OR “case-control”[Title/Abstract] OR “prospective”[Title/Abstract] OR “population-based”[Title/Abstract]) AND (“1 January 2010”[Date-Publication]: “1 June 2026”[Date-Publication]).

Both core PubMed searches were conducted on 2 June 2026, and no language restrictions were applied. To support the broader narrative scope of the two companion reviews, the core searches were supplemented, where necessary, by targeted PubMed searches and reference-list screening of key reviews and relevant articles. These supplementary procedures were used primarily for topics not fully captured by the broad risk-factor terminology of the core search strategies and were not quantified separately. Additional contextual publications released after the core search cutoff were identified during manuscript preparation and were used to frame the Introduction and Discussion.

### 2.3. Relevance Assessment and Thematic Allocation

Records retrieved from the two core PubMed searches were merged and deduplicated. Titles and abstracts were screened for relevance to either of the two companion review domains, and full texts were consulted as needed to assess thematic relevance and interpret study findings. Publications were allocated to one or both companion reviews based on the evidence they contributed to each research question. The categories were: (i) life-course determinants and primary prevention; (ii) clinical presentation, diagnostic pathways, and inherited susceptibility; or (iii) both reviews. Across the two companion reviews, 114 unique journal publications were cited. Of these, 43 were cited in both reviews, 32 only in the present life-course determinants and primary-prevention review, and 39 only in the companion review addressing clinical presentation, diagnostic pathways, and inherited susceptibility. Accordingly, the present review cited 75 journal publications. The core literature-identification process and final publication allocation are summarized in Supplementary Figure S1. The complete cross-reference and broad thematic classification of the cited journal publications are provided in Supplementary Table S1. Publications relevant to the present review addressed EOCRC, young-onset colorectal neoplasia, or related early-onset colorectal outcomes. They reported associations with modifiable exposures, metabolic or inflammatory conditions, medication use, lifestyle factors, dietary characteristics, or socioeconomic determinants. Studies of advanced adenoma or young-onset colorectal neoplasia were considered when relevant to early colorectal carcinogenesis or prevention. Publications were excluded from the present synthesis when they did not address EOCRC; did not provide information on the exposures within the scope of this review; or did not provide evidence relevant to early colorectal carcinogenesis or prevention.

### 2.4. Evidence Prioritization and Narrative Synthesis

Evidence was organized qualitatively by domain. All effect estimates reported as ORs in the present review were extracted from the cited external studies or meta-analyses and were not calculated de novo by the authors. Meta-analyses were generally presented first, followed by relevant primary observational studies that provided complementary information on exposure timing, persistence, dose–response patterns, specific exposures or clinical contexts, and comparisons with later-onset colorectal cancer. Associations based mainly on retrospective or case–control evidence were interpreted with caution, particularly where reverse causation, confounding by indication, healthcare utilization bias, residual confounding, or exposure misclassification could affect estimates. Preprints, small single-center studies, and isolated estimates of extreme effects were considered exploratory. No formal risk-of-bias assessment, certainty-of-evidence grading, or de novo quantitative pooling was performed.

## 3. Results

**Figure 1:**
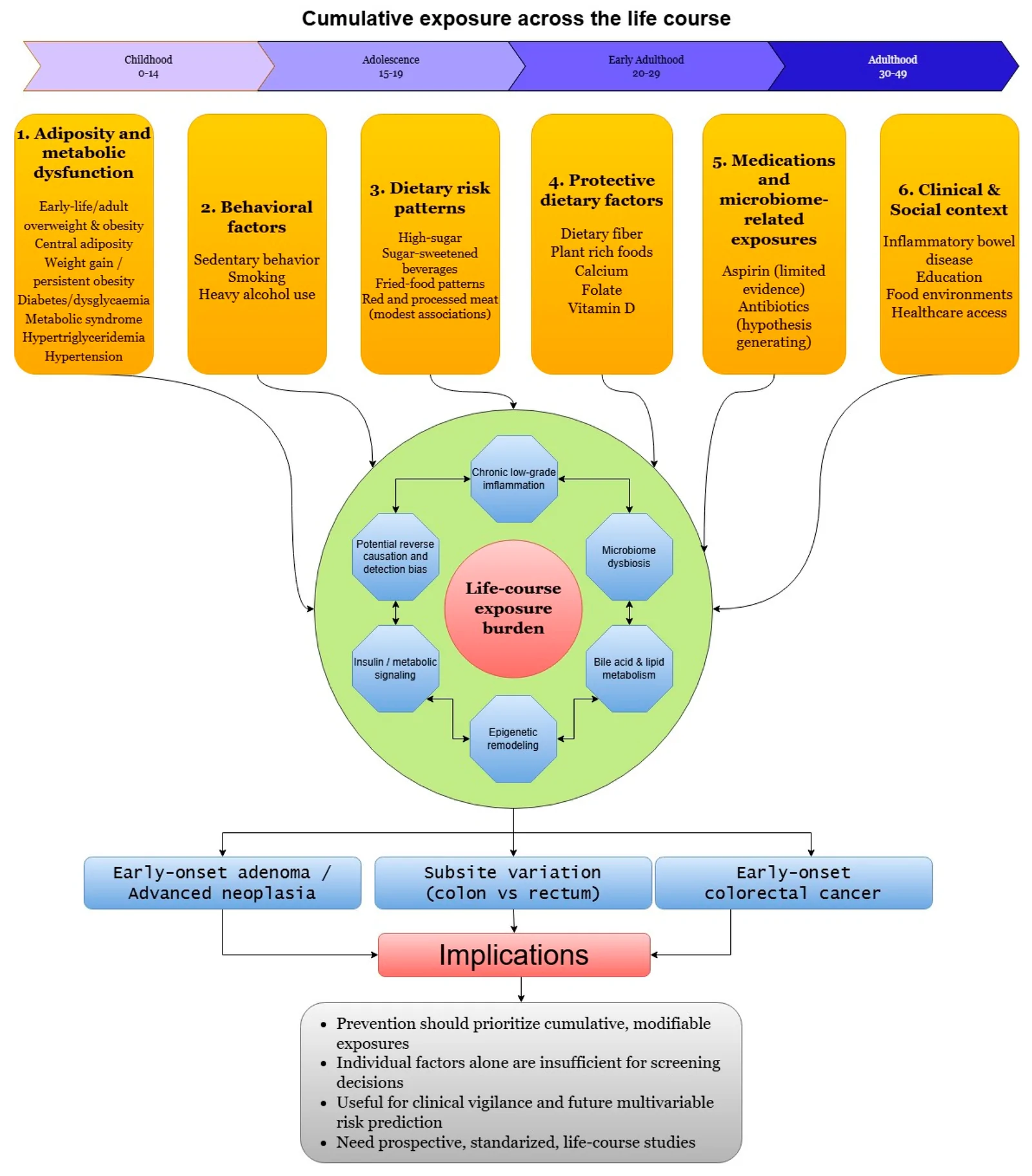
Conceptual life-course framework linking cumulative metabolic, behavioral, dietary, and medication-related exposures, clinical conditions, and socioeconomic determinants to early-onset colorectal neoplasia and colorectal cancer. Arrows represent plausible pathways and relationships inferred from the literature, rather than established causal mechanisms.

The present review drew on 75 journal publications addressing the metabolic, behavioral, dietary, medication-related, clinical, and socioeconomic determinants of EOCRC (young-onset colorectal neoplasia). Of these, 43 were also cited in the companion review, and 32 were specific to the present review. Figure 1 illustrates how cumulative exposures from childhood through adulthood may converge through metabolic, inflammatory, microbiome-related, and epigenetic pathways, with implications for prevention, clinical vigilance, and future multivariable risk prediction. Factor-specific evidence is presented in Table 1 and synthesized by domain below.

### 3.1. Adiposity Across the Life Course

Overweight, obesity, and higher BMI were generally associated with EOCRC or younger-adult colorectal cancer, although estimates varied according to study design, timing of adiposity assessment, and covariate adjustment. Pooled evidence supports a graded association, and EOCRC-focused pooled analyses also reported positive associations for adolescent overweight and elevated waist circumference [12,18]. Several primary studies further suggest that the timing and persistence of excess adiposity may be relevant [12,18,20,22,30,31,32,33,34,35,36,37,38]. Higher BMI in adolescence or early adulthood, substantial adult weight gain, and persistent or incident abdominal obesity have been associated with EOCRC in selected cohorts and case–control studies [39,40,41]. However, findings were not uniform, particularly when BMI was measured close to diagnosis or when analyses used colonoscopy-based controls, CRC-restricted comparisons, or different outcome definitions [19,24,29,42,43,44,45]. Overall, the evidence supports adiposity as a prevention-relevant component of cumulative metabolic exposure across the life course, rather than a standalone criterion for individual screening eligibility.

### 3.2. Physical Activity, Sedentary Behavior, and Sleep

Sedentary behavior was generally associated with higher EOCRC or young-onset colorectal neoplasia risk, with pooled estimates indicating ~25–33% higher odds [12,20,22]. Prolonged sitting and greater television viewing showed dose-related associations, including nearly doubled EOCRC odds for ≥10 versus <5 h/day of sitting [43,46]. Higher physical activity appeared protective in some studies, but associations were often null after adjustment, reflecting heterogeneity in exposure measurement and confounding control [21,28,29,30,33,42,47]. Sleep duration has been sparsely studied. One case–control analysis found no independent association with EOCRC risk, whereas EOCRC patients reported shorter habitual sleep than late-onset CRC patients in a case–case comparison, which does not establish incidence risk [26,48].

### 3.3. Metabolic Dysfunction and Cardiometabolic Clustering

Diabetes, dysglycaemia, and metabolic syndrome were generally associated with modestly higher EOCRC or young-onset colorectal neoplasia risk, although estimates varied across populations, outcome definitions, and adjustment strategies. Pooled EOCRC evidence suggested an association with diabetes, while a large nested case–control study reported an independent association between metabolic syndrome and EOCRC. A meta-analysis of young-onset advanced colorectal neoplasia reported a similar association for diabetes [20,22,23]. Associations were sometimes stronger in poorly controlled diabetes, with diabetic-range fasting glucose, or with more severe metabolic dysfunction, although several multivariable analyses reported attenuated or null estimates [21,24,26,28,30,31,38,42,49,50,51,52,53]. The number and combination of metabolic abnormalities appeared more informative than isolated components. In particular, EOCRC risk increased with clustering of obesity, diabetes, hypertension, and related metabolic conditions in several studies [22,30,33,54]. Hyperlipidemia and hypertension showed at most modest and heterogeneous associations: some pooled and observational analyses suggested positive associations, whereas others were null after multivariable adjustment [12,19,26,30,31,32,34,47,55]. Persistent hypertriglyceridemia may represent a more consistent lipid-related signal, particularly in selected subgroups, but requires further confirmation [56]. Overall, diabetes and metabolic syndrome are best interpreted as prevention-relevant markers within broader patterns of adiposity, insulin resistance, and cumulative cardiometabolic dysfunction, rather than as standalone criteria for individual EOCRC screening eligibility.

### 3.4. Inflammatory and Other Clinical Conditions

Inflammatory bowel disease emerged as the most consistent clinical determinant. A meta-analysis of three studies reported higher EOCRC odds among individuals with IBD (OR 3.20, 95% CI 1.60–6.41), although precision was limited by the small number of contributing studies [20]. Individual observational studies likewise reported elevated EOCRC or young-onset neoplasia risk, but the magnitude of the association varied by IBD definition, disease subtype, and comparator group [12,20,22,31,57,58,59]. Broader categories of chronic inflammatory disease and prediction-model phenotypes are less specific and should not be interpreted as equivalent to an IBD diagnosis [43,60]. Thus, IBD should be regarded as a clinically important high-risk context rather than a population-level modifiable exposure. However, timely diagnosis, effective disease control, appropriate treatment, and adherence to disease-specific surveillance represent modifiable aspects of care that may be relevant to risk management. Associations with NAFLD, diverticular disease, prior polyps, allergy/asthma, and overall comorbidity burden have been reported in isolated studies [32,43,55,61,62]. However, evidence for these secondary conditions remains sparse and heterogeneous, and some findings—particularly for diverticular disease or conditions diagnosed close to the index date—may reflect increased healthcare contact, diagnostic overlap, or reverse causation. They were therefore not prioritized as core EOCRC determinants in the present synthesis [12]. Some lifestyle and medication-related exposures considered in this review may also influence the development or activity of IBD, potentially modifying CRC risk indirectly through chronic intestinal inflammation; however, these relationships were beyond the specific scope of the present review and were not evaluated systematically.

### 3.5. Medication and Antibiotic Exposure

Aspirin use: In a large case–control study, aspirin use was associated with lower EOCRC odds [24]. However, EOCRC-specific evidence remains limited, and no pooled estimate was identified for aspirin or non-steroidal anti-inflammatory drugs as a class. Other observational analyses have reported null or inconsistent associations, and CRC-only comparisons of prediagnostic aspirin use do not directly estimate the risk of EOCRC incidence [42,43,46,63]. Thus, the observed association is consistent with the established chemopreventive role of aspirin in colorectal carcinogenesis but is insufficient to support initiating aspirin solely for EOCRC prevention.

Antibiotic exposure: Evidence was heterogeneous and may vary by tumor site, timing of exposure, indication, and antibiotic class. In a population-based case–control study including 445 EOCRC cases, any oral antibiotic use was associated with colon cancer diagnosed before age 50 (adjusted OR 1.49, 95% CI 1.07–2.07), but not with rectal cancer (adjusted OR 1.17, 95% CI 0.75–1.84); no overall exposure-response relationship was observed for antibiotic duration and early-onset colon cancer [25]. Other studies reported null overall associations or limited signals for selected broad-spectrum agents or macrolides [43,64,65,66]. These findings remain hypothesis-generating because residual confounding, confounding by indication, and healthcare utilization patterns may influence the observed associations. Evidence for other medications is sparse, heterogeneous, and largely exploratory. Reported associations for individual drug classes may reflect underlying indications, co-medication, or differences in healthcare contact and were not prioritized as core EOCRC determinants in this review [32,43,66].

### 3.6. Tobacco and Alcohol

Smoking: Cigarette smoking was generally associated with modestly increased EOCRC or advanced early-onset neoplasia risk, although findings varied by design, exposure definition, and outcome [12,19,21,22,24,26,27,29,30,31,42,43,52,53,57,58,67,68]. Current and heavy smoking showed the clearest associations, including exposure–response gradients and substantially elevated risk among heavy current smokers, whereas former, light, or pack-year–based measures were less consistently associated [21,26,29,30,42,67,68]. Several adjusted analyses reported null findings, highlighting heterogeneity and possible residual confounding [19,24,31,43,53]. A detailed smoking history may inform future multivariable EOCRC prediction models, but tobacco prevention and cessation remain the more immediate population-level priorities.

Alcohol: Alcohol exposure, particularly high intake or alcohol misuse, was generally associated with higher EOCRC risk, although null and non-linear findings were reported [12,21,26,28,29,30,31,32,33,34,42,43,47,53,57,58,69]. Pooled evidence showed increased risk for alcohol consumption, while heavier-use indicators showed stronger associations, including alcohol abuse, current drinking, ≥14 drinks/week, and 25 g/day lifetime intake [12,26,28,29,34,58,69]. Several studies supported increased risk with regular or heavy use, whereas others found no independent association or imprecise trends [21,29,30,31,32,33,42,43,47,53]. A non-linear pattern, with higher risk among both abstainers and heavy drinkers versus moderate drinkers, suggests potential confounding and exposure-definition heterogeneity [42]. These findings support the reduction in harmful alcohol consumption as a population-level prevention priority; alcohol exposure alone should not determine individual EOCRC screening eligibility.

### 3.7. High-Sugar, Sugar-Sweetened Beverage, and Fried-Food Patterns

High-sugar, sugar-sweetened beverage, and fried-food patterns showed recurrent positive associations with EOCRC or young-onset colorectal neoplasia, although dietary definitions and exposure ascertainment varied substantially across studies. A meta-analysis of young-onset advanced colorectal neoplasia found that high sugar intake was associated with higher odds of disease. A field synopsis also identified sugary beverage intake as a positive signal for EOCRC or early-onset advanced adenoma. In a multicenter Chinese case–control study, frequent consumption of sweet and fried foods (≥3 times/week) was associated with higher odds of EOCRC [22,26,27]. Some studies reported null or non-linear associations for individual sweets, desserts, or broader processed-food proxies, highlighting the limitations of isolated food categories and retrospective dietary assessment [12,22,29,43]. A sulfur microbial dietary pattern has also been associated with early-onset conventional adenoma in selected analyses, but this remains a secondary and hypothesis-generating dietary-pattern signal rather than a core EOCRC determinant [70,71]. Overall, high-sugar, sugar-sweetened beverage, and fried-food patterns represent plausible prevention-relevant targets within broader low-quality dietary environments.

### 3.8. Red and Processed Meat

Higher red-meat intake showed a small positive association with EOCRC in pooled evidence, whereas the association for processed meat was borderline under a random-effects model. In an Italian and Swiss case–control study of adults aged ≤45 years, higher processed-meat intake was associated with young-onset colorectal cancer, while red-meat intake was not [20,28]. Several case–control studies reported stronger associations at higher processed-meat intake, but null findings and between-study heterogeneity were also common [26,29,42,43]. Thus, processed meat appears to be a more consistent dietary signal than red meat. However, both associations remain modest and should be interpreted within the context of broader dietary and metabolic patterns.

### 3.9. Dietary Fiber, Plant-Rich Foods, and Micronutrient Patterns

Higher dietary fiber intake was associated with lower odds of EOCRC in a multicenter Chinese case–control study. An Italian and a Swiss case–control study also reported inverse associations with higher intake of vegetables and citrus fruits. In contrast, the association with total fruit intake was weaker and not statistically significant. Conversely, a small Italian pilot case–control study found no significant differences in vegetable, fruit, legume, or cereal consumption between EOCRC cases and controls [26,28,29]. These findings support dietary fiber and plant-rich food intake as plausible prevention-relevant signals, but EOCRC-specific evidence remains heterogeneous and relies predominantly on retrospective dietary assessment.

A field synopsis of EOCRC and early-onset advanced adenoma reported inverse associations with higher vitamin D, dietary calcium, and folate intake. Earlier case–control evidence also suggested an inverse association for folate, whereas results for calcium and vitamin D were less consistent [27,28]. These micronutrients may partly reflect broader dietary quality and correlated health-related behaviors; current evidence does not support nutrient supplementation or dairy consumption as stand-alone EOCRC-prevention strategies. Evidence for individual foods and beverages not retained in Table 1 was sparse, inconsistent, or based on isolated case–control studies.

### 3.10. Socioeconomic Context

Socioeconomic associations with EOCRC are heterogeneous. Lower education was associated with higher EOCRC risk in some studies, and higher education was strongly protective in one analysis, whereas other studies found no significant association [21,26,28,42]. Income and access-related indicators were also inconsistent: some analyses found no association with income or lower EOCRC odds in the lowest-income quartile, whereas others linked lower socioeconomic status, insurance characteristics, cohabitation, and employment patterns to EOCRC risk [21,31,32,72]. At the contextual level, EOCRC burden varies by socio-demographic index, with stronger contributions from Westernized diet, obesity, and metabolic risks in high- and middle-SDI regions, while urban/rural residence was not independently associated in one study [13,53]. Lower socioeconomic background was also associated with poorer recognition of CRC risk factors and warning signs and greater endorsement of inaccurate causes [73]. Educational attainment, healthcare access, and area-level socioeconomic conditions are best understood as contextual determinants that shape exposure patterns, health literacy, and opportunities for prevention and timely evaluation, rather than as individual markers for EOCRC screening eligibility.

## 4. Discussion

The evidence synthesized in this review is compatible with a life-course model of EOCRC in which risk may be shaped by the timing, persistence, and accumulation of multiple exposures rather than by a single determinant. Evidence was most consistent for early and sustained adiposity and cardiometabolic dysfunction, while IBD emerged as a strong but clinically distinct high-risk condition. Sedentary behavior and higher alcohol intake showed recurrent but heterogeneous associations, whereas findings for smoking, dietary factors, medications, and antibiotics were less consistent. Potentially inverse associations for dietary fiber, plant-rich foods, calcium, folate, and vitamin D may partly reflect broader dietary quality and correlated health-related behaviors rather than isolated nutrient effects. These factors frequently coexist and may reinforce one another through shared metabolic, inflammatory, behavioral, and socioeconomic pathways [12,20,22,23,24,26,28,37,54].

A formal ranking of the ten factors was not considered appropriate due to substantial heterogeneity in study designs, exposure definitions, outcomes, and effect estimates, and the absence of a de novo quantitative synthesis. Instead, the evidence was qualitatively differentiated according to consistency and clinical context, with excess adiposity and cardiometabolic dysfunction showing the most consistent associations, IBD representing a strong but clinically distinct high-risk condition, and other factors showing more heterogeneous or limited evidence.

Other determinants showed more limited or heterogeneous evidence but remain relevant within the broader life-course framework. Smoking was associated with EOCRC in some studies but showed inconsistent pooled estimates. Dietary components such as fiber, calcium, folate, and vitamin D showed possible inverse associations that may partly reflect overall dietary quality rather than isolated effects. Medication and antibiotic associations were limited and vulnerable to confounding. At the same time, socioeconomic conditions appeared to shape exposure patterns, healthcare access, and opportunities for prevention rather than to act as independent risk markers.

The life-course perspective is particularly relevant because exposures beginning during childhood, adolescence, or early adulthood may act over a longer prediagnostic period. Early overweight, persistent obesity, weight gain, dysglycaemia, sedentary behavior, and unhealthy dietary patterns may therefore be more informative than a single contemporaneous measurement. This framework is compatible with birth-cohort patterns suggesting that successive generations have experienced earlier and more sustained exposure to obesogenic environments, changing food systems, reduced physical activity, tobacco and alcohol use, and broader socioeconomic transitions [2,11].

However, EOCRC should not be portrayed as a disease caused by a unique set of environmental exposures. Pooled analyses of non-genetic determinants generally report effect sizes broadly comparable with those observed in later-onset CRC for many established factors. These include adiposity, diabetes, smoking, alcohol use, dietary quality, and inflammatory conditions [42]. One plausible but incompletely tested explanation is that familiar colorectal cancer determinants begin earlier, persist longer, or interact cumulatively before midlife. This age divergence may partly reflect the preventive effects of organized screening and removal of premalignant lesions in older populations. Nevertheless, it should prompt proportionate action rather than alarm, without obscuring the substantially greater absolute burden of CRC among older adults [74]. This interpretation may also help explain heterogeneity by sex, population, tumor subsite, and healthcare context. Direct comparisons between EOCRC and later-onset CRC remain limited, and future studies should determine whether particular exposures exert genuinely stronger effects in younger adults or primarily accelerate carcinogenic processes that also operate later in life.

The associations reviewed here should not be interpreted as proof of causality. Much of the EOCRC literature is based on retrospective case–control designs, self-reported dietary and lifestyle measures, and exposure assessment close to diagnosis. Recall bias, residual confounding, reverse causation, healthcare utilization bias, and confounding by indication are therefore important concerns, particularly for diet, body weight, medication use, and antibiotic use. Nevertheless, the clustering of excess adiposity, insulin resistance, altered lipid metabolism, and chronic low-grade inflammation provides biological plausibility for several of the observed patterns [23,27,46,50]. Diet, sedentary behavior, alcohol exposure, and metabolic dysfunction may converge through related inflammatory and metabolic pathways. IBD represents a clinically distinct example of chronic inflammation associated with substantially elevated colorectal-cancer risk [20]. Microbiome-related mechanisms may help connect diet, medication exposure, and inflammation, but direct EOCRC-specific evidence remains limited; antibiotics, dysbiosis, and other emerging environmental hypotheses should therefore be regarded as hypothesis-generating rather than established explanations. Human studies specifically addressing the microbiome in EOCRC remain scarce, and available evidence is fragmented across small observational datasets and heterogeneous microbial measures. Accordingly, microbiome-related pathways shown in Figure 1 should be interpreted as plausible mechanistic links rather than established EOCRC-specific causal pathways, pending prospective human studies with longitudinal microbiome assessment.

These findings support prevention strategies that address cumulative exposures throughout the life course. Population priorities include prevention of early and persistent excess adiposity, reduction in sedentary behavior, tobacco prevention and cessation, reduction in harmful alcohol consumption, and improvement of food environments that facilitate frequent intake of processed meat, added sugars, sugar-sweetened beverages, and fried foods. Such measures are unlikely to prevent EOCRC in isolation, but may reduce the cumulative burden of colorectal cancer determinants while improving broader metabolic and cardiovascular health. Educational attainment and socioeconomic position should be interpreted as contextual markers of differences in food environments, health literacy, healthcare use, and access to prevention, rather than as independent individual risk markers. Individual factors remain insufficient to determine screening eligibility in younger adults, and lowering the screening age alone is unlikely to provide a complete response. More proportionate approaches may eventually integrate multiple domains within multivariable risk-prediction models. These may include age, symptoms, anemia-related findings, life-course exposures, metabolic conditions, family history, inherited susceptibility, demographic variables, and healthcare context within multivariable risk-prediction models [74]. IBD warrants separate consideration as a high-risk clinical condition requiring optimal inflammatory control, adherence to disease-specific surveillance, and timely assessment of changing symptoms.

These findings also have implications for European cancer-prevention policy. The concentration of EOCRC-associated exposures across successive birth cohorts supports life-course measures that promote healthier food environments, prevent persistent adiposity and metabolic dysfunction, reduce sedentary behavior, strengthen tobacco control, and reduce harmful alcohol consumption. These priorities are aligned with the prevention objectives of Europe’s Beating Cancer Plan [75], the European Code Against Cancer [76], and the Farm to Fork Strategy [77]. Within the ONCODIR Work Package 2 framework, this synthesis provides a bridge between epidemiological evidence, prevention-oriented policy, and equitable implementation across European settings. Together with the companion review on clinical presentation, diagnostic pathways, and inherited susceptibility, it supports a complementary framework in which the present review addresses primary prevention before neoplasia develops. In contrast, the companion paper focuses on earlier detection and individualized clinical-genetic assessment.

### Strengths and Limitations

This narrative review used two prespecified PubMed strategies targeting meta-analyses and observational EOCRC studies published from 1 January 2010 through 1 June 2026. Its main strength is the structured synthesis of potentially modifiable exposures, clinical risk conditions, and socioeconomic or contextual determinants across EOCRC, young-onset colorectal neoplasia, and related outcomes. This approach distinguishes the more consistent evidence for excess adiposity and cardiometabolic dysfunction, the clinically distinct high-risk context of IBD, recurrent but heterogeneous associations for sedentary behavior and higher alcohol intake, and more limited or heterogeneous findings for smoking, dietary factors, medications, and antibiotics. Several limitations should be acknowledged. Although literature identification and thematic allocation followed a structured process documented in Supplementary Figure S1 and Supplementary Table S1, this work was designed as a narrative rather than a systematic review. The core searches were restricted to PubMed. They were supplemented by targeted PubMed searches and reference-list screening that were not separately quantified. Accordingly, the flow diagram summarizes the core literature-identification process and the final allocation of cited journal publications rather than an exhaustive systematic-review selection pathway. No formal risk-of-bias assessment, certainty-of-evidence grading, or de novo quantitative pooling was performed, and relevant studies not captured by the search terminology or supplementary identification procedures may have been missed. The underlying studies differed in EOCRC definitions, exposure measurement, assessment timing, covariate adjustment, comparator groups, and outcome definitions. Many associations were evaluated in relatively few EOCRC-specific datasets and may not be directly transferable across populations. Reverse causation, recall bias, residual confounding, confounding by indication, and healthcare utilization bias are particularly relevant to medication exposure, antibiotic use, body weight, diet, and prediagnostic lifestyle changes. Finally, the review was deliberately restricted to prevention-relevant exposures, clinical risk conditions, and contextual determinants; clinical presentation, diagnostic pathways, family history, and inherited susceptibility are addressed separately in the companion review. Restricting core literature identification to PubMed may also have limited the capture of relevant evidence published outside the PubMed-indexed literature, including gray literature and regional sources, despite the absence of language restrictions. Accordingly, some relevant evidence may not have been captured by the core search and supplementary identification procedures.

## 5. Conclusions

Evidence was most consistent for excess adiposity and cardiometabolic dysfunction, while IBD emerged as a clinically distinct high-risk condition. Sedentary behavior, smoking, higher alcohol intake, and lower-quality dietary patterns showed recurrent but heterogeneous associations. Associations involving dietary and antibiotic exposures remain predominantly observational and are subject to residual confounding, limiting causal inference. Earlier exposure, longer duration, and cumulative interaction of familiar CRC determinants remain plausible but incompletely tested explanations rather than established distinctive features of EOCRC. The findings support population-level primary prevention through healthier food and activity environments, prevention of persistent adiposity and metabolic dysfunction, tobacco control, and reduced harmful alcohol consumption. No individual factor should determine screening eligibility in younger adults. Rather, prospective life-course studies are needed to inform future multivariable models. This review complements the companion synthesis on clinical presentation, diagnostic pathways, and inherited susceptibility by focusing on prevention before symptoms or neoplasia develop.

## Figures and Tables

**Table 1:** Modifiable, clinical, and socioeconomic determinants associated with early-onset colorectal cancer or young-onset colorectal neoplasia: a critical narrative summary of prevention-relevant evidence.

Domain	Exposure or Determinant	Key Finding	Interpretation for Primary Prevention or Clinical Context	Key Refs.
Anthropometric and life-course factors	Adiposity across the life course.	Pooled evidence supports a graded association between BMI and EOCRC or younger-adult CRC: compared with normal BMI, overweight and obesity were associated with ORs of 1.32 (95% CI 1.19–1.47) and 1.88 (95% CI 1.40–2.54), respectively. EOCRC-focused pooled analyses also found positive associations for adolescent overweight (OR 1.37, 95% CI 1.15–1.63) and elevated waist circumference (OR 1.17, 95% CI 1.01–1.34).	Excess adiposity is among the more consistent modifiable associations, particularly when overweight begins early or persists over time. The findings support population-level prevention of early and sustained weight gain; however, BMI or waist measures should not be used alone to determine individual screening eligibility.	[12,18,19]
Sedentary behavior and physical activity	Sedentary behavior and physical activity	A meta-analysis of five studies found that sedentary behavior was associated with higher odds of EOCRC (OR 1.33, 95% CI 1.05–1.70). In contrast, a pooled analysis of two Canadian prospective cohorts found no statistically significant association between high versus low physical activity and EOCRC incidence (HR 1.18, 95% CI 0.70–1.98).	Sedentary behavior shows a more consistent adverse association than physical activity shows a protective association. Reducing prolonged sedentary time and promoting regular physical activity remain appropriate population-level prevention goals, but this domain is not sufficiently specific to determine individual screening eligibility.	[20,21]
Cardiometabolic factors	Diabetes, dysglycaemia, and metabolic syndrome	Pooled EOCRC evidence suggests a modest association with diabetes (OR 1.25, 95% CI 1.04–1.50). In a large nested case–control study, metabolic syndrome was associated with EOCRC after adjustment for healthcare utilization and major confounders (OR 1.25, 95% CI 1.09–1.43). A meta-analysis of young-onset advanced colorectal neoplasia reported a similar association for diabetes (OR 1.23, 95% CI 1.15–1.32).	Diabetes and metabolic syndrome represent prevention-relevant cardiometabolic signals, although the available evidence does not establish independent causal effects for each component. Their relevance is likely greatest within broader patterns of adiposity, insulin resistance, and cumulative metabolic dysfunction.	[20,22,23]
Cardiometabolic factors	Cardiometabolic clustering and metabolic comorbidities	In the same large EOCRC study, risk increased progressively with the number of metabolic comorbidities. Compared with no metabolic conditions, adjusted ORs were 1.09 (95% CI 1.00–1.17), 1.12 (95% CI 1.01–1.24), and 1.31 (95% CI 1.13–1.51) for one, two, and three or more conditions, respectively. Individual hypertension and hyperlipidemia estimates have been heterogeneous or non-significant in pooled analyses.	The overall cardiometabolic profile appears more informative than isolated blood-pressure or lipid abnormalities. Prevention should address clustering of excess adiposity, dysglycaemia, dyslipidemia, and hypertension, rather than treating any single component as a standalone EOCRC marker.	[12,23]
Clinical conditions	Inflammatory bowel disease	A meta-analysis of three studies found that inflammatory bowel disease was associated with higher odds of EOCRC (OR 3.20, 95% CI 1.60–6.41). The estimate indicates a substantial association, although the small number of contributing studies limits its precision.	Inflammatory bowel disease is a clinically important high-risk condition rather than a directly modifiable population-level exposure. However, timely diagnosis, effective disease control, appropriate treatment, and adherence to disease-specific surveillance may represent modifiable aspects of care relevant to risk management.	[20]
Medication exposure	Aspirin use	In a large case–control study, aspirin use was associated with lower odds of EOCRC (OR 0.70, 95% CI 0.50–0.80). However, EOCRC-specific evidence remains limited, and no pooled estimate was identified for aspirin or non-steroidal anti-inflammatory drugs as a class.	The observed association is consistent with the established chemopreventive role of aspirin in colorectal carcinogenesis, but it is insufficient to support initiating aspirin solely for the prevention of EOCRC. Any potential benefit must be weighed against bleeding risk and considered within established clinical indications and guideline-based risk–benefit assessment.	[24]
Medication exposure	Antibiotics	In a population-based case–control study including 445 EOCRC cases, any oral antibiotic use was associated with colon cancer diagnosed before age 50 (adjusted OR 1.49, 95% CI 1.07–2.07), but not with rectal cancer (adjusted OR 1.17, 95% CI 0.75–1.84). No overall exposure-response relationship was observed for antibiotic duration and early-onset colon cancer.	This is a hypothesis-generating, site-specific association based on a single observational study. Residual confounding, confounding by indication, and limited EOCRC case numbers preclude causal inference. Antibiotic prescribing should continue to follow established clinical indications and antimicrobial stewardship principles; current evidence does not support using prior antibiotic exposure to guide individual EOCRC screening.	[25]
Behavioral factors	Cigarette smoking	In a multicentre Chinese case–control study, current smoking was associated with higher EOCRC odds compared with never smoking (OR 1.67, 95% CI 1.08–2.58), whereas former smoking was not significantly associated. Pooled EOCRC evidence has been heterogeneous: a meta-analysis of EOCRC studies reported a non-significant random-effects estimate (OR 1.23, 95% CI 0.93–1.64), while a meta-analysis of young-onset advanced colorectal neoplasia found a modest positive association (OR 1.19, 95% CI 1.05–1.36).	Smoking is a modifiable exposure with an established role in colorectal carcinogenesis. EOCRC-specific evidence is suggestive but heterogeneous, and smoking status should not be used alone for individual risk stratification. Smoking prevention and cessation remain appropriate population-level cancer-prevention priorities.	[20,22,26]
Behavioral factors	High alcohol intake	A meta-analysis of five EOCRC studies found that higher alcohol consumption was associated with increased EOCRC odds compared with lower consumption (OR 1.52, 95% CI 1.28–1.80). In a multicentre Chinese case–control study, current drinking was also associated with higher EOCRC odds versus never drinking (OR 1.63, 95% CI 1.07–2.48), whereas ever drinking was not significantly associated. A meta-analysis of young-onset advanced colorectal neoplasia reported a similar association for high alcohol intake (OR 1.37, 95% CI 1.10–1.71).	Higher alcohol intake is a relatively consistent modifiable association, although studies differ in exposure definitions, drinking patterns, and adjustment for smoking and other lifestyle factors. Reducing harmful alcohol consumption is an appropriate population-level cancer-prevention priority, but alcohol exposure alone should not determine individual EOCRC screening eligibility.	[20,22,26]
Dietary patterns	High-sugar, sugar-sweetened beverage, and fried-food patterns	A meta-analysis of young-onset advanced colorectal neoplasia found that high sugar intake was associated with higher odds of disease (OR 2.58, 95% CI 1.61–4.13). A field synopsis also identified sugary beverage intake as a positive signal for EOCRC or early-onset advanced adenoma. In a multicentre Chinese case–control study, frequent consumption of sweet foods and fried foods (≥3 times/week) was associated with higher EOCRC odds (OR 2.70, 95% CI 1.89–3.86 and OR 2.16, 95% CI 1.29–3.62, respectively). However, dietary definitions and exposure ascertainment varied substantially across studies.	High-sugar and fried-food patterns are plausible prevention-relevant dietary targets, particularly as components of broader low-quality or highly processed dietary environments. The evidence supports reducing sugar-sweetened beverages, added sugars, and frequent fried-food intake as part of overall dietary improvement, but individual food categories should not be used alone for EOCRC risk stratification.	[22,26,27]
Dietary components	Red and processed meat	A meta-analysis of EOCRC studies found a small positive association for higher red-meat intake (OR 1.12, 95% CI 1.07–1.17), whereas the association for processed meat was borderline under a random-effects model (OR 1.24, 95% CI 0.99–1.55). In an Italian and Swiss case–control study of adults aged ≤45 years, higher processed-meat intake was associated with young-onset colorectal cancer, while red-meat intake was not.	Meat-related associations appear modest and not fully consistent across EOCRC studies. Reducing processed-meat intake and moderating red-meat consumption are consistent with broader colorectal-cancer prevention guidance, but neither exposure should be used alone for individual EOCRC risk stratification.	[20,28]
Dietary components	Dietary fiber and plant-rich food intake	In a multicentre Chinese case–control study, higher dietary-fiber intake was associated with lower EOCRC odds (OR 0.53, 95% CI 0.33–0.85). In an Italian and Swiss case–control study, higher intake of vegetables and citrus fruits was inversely associated with young-onset colorectal cancer. In contrast, the association for total fruit intake was weaker and not statistically significant. Conversely, a small Italian pilot case–control study found no significant differences in vegetable, fruit, legume, or cereal consumption between EOCRC cases and controls.	Dietary fiber and plant-rich food intake represent plausible prevention-relevant signals, but EOCRC-specific evidence remains heterogeneous and largely relies on retrospective dietary assessments. These findings support overall improvements in dietary quality rather than attributing risk or protection to individual foods or dietary labels.	[26,28,29]
Dietary components	Calcium, folate, and vitamin D intake	A field synopsis of EOCRC and early-onset advanced adenoma reported inverse associations for higher vitamin D intake (OR 0.75, 95% CI 0.59–0.95), dietary calcium intake (OR 0.82, 95% CI 0.68–0.99), and folate intake (OR 0.77, 95% CI 0.60–0.99). Earlier case–control evidence also suggested an inverse association for folate, whereas results for calcium and vitamin D were less consistent.	These micronutrients may reflect broader dietary quality and are potentially relevant to colorectal-cancer prevention. However, current EOCRC-specific evidence does not justify nutrient supplementation or dairy consumption as stand-alone preventive strategies; findings require confirmation in prospective studies with better characterization of food sources and overall dietary patterns.	[27,28]
Socioeconomic context	Educational attainment and socioeconomic context	In a multicentre Chinese case–control study, higher educational attainment was inversely associated with EOCRC in a multivariable prediction model (OR 0.48, 95% CI 0.40–0.58). However, pooled EOCRC evidence for educational level has been heterogeneous, and a European case–control study found no statistically significant association. Educational attainment may therefore capture broader differences in socioeconomic resources, diet, health literacy, healthcare utilization, and access to prevention rather than an independent causal exposure.	Education should be regarded as a contextual social determinant rather than an individual EOCRC risk marker. The available evidence supports equitable prevention, health literacy, access to healthy environments, and timely healthcare evaluation, but educational level should not be used alone to assign individual screening eligibility.	[20,26,28]

Abbreviations: BMI, body mass index; CI, confidence interval; CRC, colorectal cancer; EOCRC, early-onset colorectal cancer; HR, hazard ratio; OR, odds ratio; Refs., references.

## Data Availability

No new data were created or analyzed in this study. Data sharing is not applicable to this article.

## Supplementary Materials

The following supporting information can be downloaded at https://www.mdpi.com/article/10.3390/cancers18172852/s1, Figure S1: Flow diagram of literature identification, screening, eligibility assessment, and inclusion in the narrative synthesis; Table S1: Cross-reference and thematic classification of journal publications cited in the two companion narrative reviews.
