# Supplementary material for: Understanding the Rising Incidence of Early-Onset Colorectal Cancer: Life-Course Determinants and Opportunities for Primary Prevention
Source: Cancers (Basel). 2026 Sep 3;18(17):2852. doi: 10.3390/cancers18172852 (PMC13564976; doi:10.3390/cancers18172852)
Supplement: Supplementary file 1 [file cancers-18-02852-s001.zip › Supplementary Table S1. Cross-reference and thematic classification of journal publications cited in the two companion narrative reviews.pdf]

Supplementary Table S1. Cross-reference and thematic classification of journal publications cited in the two companion narrative reviews.

Paper	*Cited in both companion reviews*	*Cited only in the life-course determinants and primary-prevention review*	*Cited only in the clinical presentation, diagnostic pathways, and inherited-susceptibility review*	*Topic*
Abdel-Rahman et al., 2022—Patterns of colorectal cancer diagnosis among younger adults in a real-world, population-based cohort [71]	✓			Clinical/anatomical phenotype
Abu-Freha et al., 2025—Early onset colorectal cancer, not just the age: Data from a large health organization [44]	✓			Clinical/anatomical phenotype
Akimoto et al., 2021—Rising incidence of early-onset colorectal cancer - a call to action [45]	✓			Overview and emerging mechanisms
Alshenaifi et al., 2025—Mutational and co-mutational landscape of early onset colorectal cancer [47]	✓			Molecular and tumor biology
Berrington de Gonzalez et al., 2025—Trends in cancer incidence in younger and older adults: an international comparative analysis [7]	✓			Epidemiology and incidence trends
Bolwell et al., 2024—Risk Factors Associated with Advanced Colorectal Neoplasia in Adults Younger than Age 45 [51]	✓			Mixed risk factors and prediction
Brenner et al., 2019—National Trends in Colorectal Cancer Incidence Among Older and Younger Adults in Canada [3]	✓			Epidemiology and incidence trends

Cann et al., 2025—Generations at Risk: Unveiling the Global Increase in Cancer at Both Ends of Adulthood [15]	✓	Overview and emerging mechanisms
Chang et al., 2021—Risk factors for early-onset colorectal cancer: a population-based case–control study in Ontario, Canada [42]	✓	Mixed risk factors and prediction
Chen et al., 2022—Alcohol consumption, polygenic risk score, and early- and late-onset colorectal cancer risk [68]	✓	Tobacco and alcohol
Chen et al., 2024—Comparing cohort and period trends of early-onset colorectal cancer: a global analysis [10]	✓	Epidemiology and incidence trends
Danial et al., 2022—Risk Factors of Young-Onset Colorectal Cancer: Analysis of a Large Population-Based Registry [57]	✓	Mixed risk factors and prediction
Deng et al., 2023—Noninvasive predictive models based on lifestyle analysis and risk factors for early-onset colorectal cancer [25]	✓	Mixed risk factors and prediction
Erdem et al., 2025—Snapshot cohort study on early-onset colorectal cancer in Northwestern Switzerland: are we doing enough or can we do more? [62]	✓	Clinical/anatomical phenotype
Exarchakou et al., 2019—Colorectal cancer incidence among young adults in England: Trends by anatomical sub-site and deprivation [9]	✓	Socioeconomic/demographic disparities
Gu et al., 2022—A risk scoring system to predict the individual incidence of early-onset colorectal cancer [19]	✓	Mixed risk factors and prediction

Hayes, 2021—Advances in Understanding Early-Onset Colorectal Cancer [1]	✓	Overview and emerging mechanisms
Imperiale et al., 2023—Risk Factors for Early-onset Sporadic Colorectal Cancer in Male Veterans [31]	✓	Mixed risk factors and prediction
Khan et al., 2015—Dietary Practices, Addictive Behavior and Bowel Habits and Risk of Early Onset Colorectal Cancer: a Case Control Study [66]	✓	Diet and nutrition
Kim et al., 2016—Different risk factors for advanced colorectal neoplasm in young adults [46]	✓	Mixed risk factors and prediction
Low et al., 2020—Risk Factors for Early-Onset Colorectal Cancer [23]	✓	Mixed risk factors and prediction
O'Sullivan et al., 2022—Risk Factors for Early-Onset Colorectal Cancer: A Systematic Review and Meta-analysis [33]	✓	Mixed risk factors and prediction
O'Sullivan et al., 2024—Risk factors for early-onset colorectal cancer: A Canadian prospective cohort study [20]	✓	Mixed risk factors and prediction
Pan et al., 2023—Risk factors for early-onset colorectal cancer: A large-scale Chinese cohort study [52]	✓	Mixed risk factors and prediction
Park et al., 2022—Clinical features of young onset colorectal cancer patients from a large cohort at a single cancer center [43]	✓	Clinical/anatomical phenotype
Rosato et al., 2013—Risk factors for young-onset colorectal cancer [27]	✓	Mixed risk factors and prediction
Ruiz-Grajales et al., 2024—Five-year overall survival of early- and late-onset colorectal cancer in Medellín, Colombia: a comparative study [14]	✓	Prognosis/surveillance/management

Sandhu et al., 2025—Evaluating Risk Factors for Early-Onset Colorectal Cancer in a Large, Prospective Cohort [18]	✓		Mixed risk factors and prediction
Saraiva et al., 2023—Early-onset colorectal cancer: A review of current knowledge [6]	✓		Overview and emerging mechanisms
Shen et al., 2021—Risk Factors Associated With Early-Onset Colorectal Neoplasm in Chinese Youth: A Prospective Population-Based Study [34]	✓		Mixed risk factors and prediction
Siegel et al., 2023—Colorectal cancer statistics, 2023 [2]	✓		Epidemiology and incidence trends
Silva et al., 2019—Young-age onset colorectal cancer in Brazil: Analysis of incidence, clinical features, and outcomes in a tertiary cancer center [4]	✓		Epidemiology and incidence trends
Sun et al., 2025—Predicting Early-Onset Colorectal Cancer in Individuals Below Screening Age Using Machine Learning and Real-World Data: Case Control Study [59]	✓		Mixed risk factors and prediction
Syed et al., 2019—Old vs new: Risk factors predicting early onset colorectal cancer [54]	✓		Mixed risk factors and prediction
Tait et al., 2023—Early-Onset Colorectal Cancer: Prevalence, Risk Factors, and Clinical Features Among Commercially Insured Adults in the United States [56]	✓		Clinical/anatomical phenotype
Tang et al., 2023—Global burden of early-onset colorectal cancer among people aged 40-49 years from 1990 to 2019 and predictions to 2030 [32]	✓		Epidemiology and incidence trends

Tang et al., 2024—Molecular characteristics of early-onset compared with late-onset colorectal cancer: a case controlled study [29]	✓		Molecular and tumor biology
The Lancet, 2026—Cancer in young adults: out of the shadows? [8]	✓		Overview and emerging mechanisms
Tian et al., 2026—Biological aging and generational shifts in early-onset cancer risk [16]	✓		Overview and emerging mechanisms
Urback et al., 2024—Serum ferritin and the risk of early-onset colorectal cancer [30]	✓		Symptoms and diagnostic pathways
Willauer et al., 2019—Clinical and molecular characterization of early-onset colorectal cancer [5]	✓		Molecular and tumor biology
Ye et al., 2023—Risk factors for advanced colorectal neoplasm in young adults: a meta-analysis [21]	✓		Mixed risk factors and prediction
Zhang et al., 2023—Field Synopsis of Environmental and Genetic Risk Factors of Sporadic Early-Onset Colorectal Cancer and Advanced Adenoma [12]	✓		Mixed risk factors and prediction
Albreht, 2021—Europe's beating cancer plan-a new step towards more comprehensive and equitable cancer control in Europe [74]		✓	Policy and primary prevention
Archambault et al., 2021—Nongenetic Determinants of Risk for Early-Onset Colorectal Cancer [41]		✓	Mixed risk factors and prediction
Ben-Aharon et al., 2023—Pharmaceutical Agents as Potential Drivers in the Development of Early-Onset Colorectal Cancer: Case-Control Study [65]		✓	Medications and antibiotics

Carroll et al., 2022—Diet as a Risk Factor for Early-Onset Colorectal Adenoma and Carcinoma: A Systematic Review [70]	✓	Diet and nutrition
Chang et al., 2024—The Persistence of Hypertriglyceridemia and the Risk of Early Onset Colorectal Cancer According to Tumor Subsites: A Nationwide Population-Based Study [55]	✓	Cardiometabolic factors
Chen et al., 2021—Metabolic syndrome, metabolic comorbid conditions and risk of early-onset colorectal cancer [22]	✓	Cardiometabolic factors
Galicia Pacheco et al., 2024—Socio-Economic Inequalities in Beliefs About Cancer and its Causes: Evidence From two Population Surveys [72]	✓	Socioeconomic/demographic disparities
Gong et al., 2025—Epidemiological trends of early-onset gastrointestinal cancers from 1990 to 2021 and predictions for 2036: analysis from the global burden of disease study 2021 [35]	✓	Epidemiology and incidence trends
Hidayat et al., 2018—Body fatness at an early age and risk of colorectal cancer [36]	✓	Adiposity and weight trajectories
Jin et al., 2022—Association Between Metabolic Syndrome and the Risk of Colorectal Cancer Diagnosed Before Age 50 Years According to Tumor Location [53]	✓	Cardiometabolic factors

Kane et al., 2024—Oral Antibiotic Use in Adulthood and Risk of Early-Onset Colorectal Cancer: A Case-Control Study [64]	✓	Medications and antibiotics
Khoa Ta et al., 2023—Association of diabetes mellitus with early-onset colorectal cancer: A systematic review and meta-analysis of 19 studies including 10 million individuals and 30,000 events [48]	✓	Cardiometabolic factors
Kim et al., 2025—Association Between Nonalcoholic Fatty Liver Disease and Risk of Early-onset Colorectal Cancer [60]	✓	Cardiometabolic factors
Li et al., 2021—Association of Body Mass Index With Risk of Early-Onset Colorectal Cancer: Systematic Review and Meta-Analysis [17]	✓	Adiposity and weight trajectories
Li et al., 2022—Associations of Body Mass Index at Different Ages With Early-Onset Colorectal Cancer [38]	✓	Adiposity and weight trajectories
Li et al., 2022—Type 2 Diabetes and Risk of Early-Onset Colorectal Cancer [49]	✓	Cardiometabolic factors
Li et al., 2023—Associations of smoking with early- and late-onset colorectal cancer [67]	✓	Tobacco and alcohol
Liu et al., 2019—Association of Obesity With Risk of Early-Onset Colorectal Cancer Among Women [39]	✓	Adiposity and weight trajectories

Lundqvist et al., 2023—Autoimmune and Metabolic Diseases and the Risk of Early-Onset Colorectal Cancer, a Nationwide Nested Case-Control Study [58]	✓	Inflammatory and clinical conditions
Luo et al., 2024—Associations between blood glucose and early- and late-onset colorectal cancer: evidence from two prospective cohorts and Mendelian randomization analyses [50]	✓	Cardiometabolic factors
McDowell et al., 2022—Oral antibiotic use and early-onset colorectal cancer: findings from a case-control study using a national clinical database [24]	✓	Medications and antibiotics
Nguyen et al., 2021—The Sulfur Microbial Diet Is Associated With Increased Risk of Early-Onset Colorectal Cancer Precursors [69]	✓	Diet and nutrition
Nguyen et al., 2022—Antibiotic Therapy and Risk of Early-Onset Colorectal Cancer: A National Case-Control Study [63]	✓	Medications and antibiotics
Ni et al., 2023—Elucidating the Drivers for the Rising Incidence of Early-Onset Colorectal Cancer: How Ecologic Studies Could Help and What Is Next [11]	✓	Overview and emerging mechanisms
Norat et al., 2015—European code against cancer 4th edition: diet and cancer [75]	✓	Policy and primary prevention
Puzzono et al., 2022—Diet and Lifestyle Habits in Early-Onset Colorectal Cancer: A Pilot Case-Control Study [28]	✓	Diet and nutrition

Schebesta et al., 2020—The European union farm to fork strategy [76]	✓		Policy and primary prevention
Schumacher et al., 2021—Metabolic Risk Factors Associated with Early-Onset Colorectal Adenocarcinoma: A Case-Control Study at Kaiser Permanente Southern California [37]	✓		Cardiometabolic factors
Song et al., 2023—Association of changes in obesity and abdominal obesity status with early-onset colorectal cancer risk: a nationwide population-based cohort study [40]	✓		Adiposity and weight trajectories
Wang et al., 2022—Time Trend and Association of Early-Onset Colorectal Cancer with Diverticular Disease in the United States: 2010-2021 [61]	✓		Inflammatory and clinical conditions
Wang et al., 2025—Global Epidemiology of Early-Onset Digestive System Malignancy: A Systematic Analysis for the Global Burden of Disease Study 2021 [13]	✓		Epidemiology and incidence trends
Zhang et al., 2023—Knowledge mapping of early-onset colorectal cancer from 2000 to 2022: A bibliometric analysis [26]	✓		Overview and emerging mechanisms
Alsiary et al., 2023—Early-Onset Colorectal Cancer Survival among Unscreened Population - Multicenter Cohort Retrospective Analysis		✓	Prognosis/surveillance/management
Archambault et al., 2020—Cumulative Burden of Colorectal Cancer–Associated Genetic Variants Is More Strongly Associated With Early-Onset vs Late-Onset Cancer		✓	Hereditary and genetic susceptibility

Baronas et al., 2024—Symptom Burden and Time from Symptom Onset to Cancer Diagnosis in Patients with Early-Onset Colorectal Cancer: A Multicenter Retrospective Analysis	✓	Symptoms and diagnostic pathways
Barot et al., 2025—Incidence trends and long-term survival in early-onset colorectal cancer: a nationwide Swedish study	✓	Epidemiology and incidence trends
Brim et al., 2025—Trends and Symptoms Among Increasing Proportion of African Americans with Early-Onset Colorectal Cancer over a 60-Year Period	✓	Socioeconomic/demographic disparities
Cavestro Giulia et al., 2022—Delphi initiative for early-onset colorectal cancer (DIRECt). International Management Guidelines	✓	Hereditary and genetic susceptibility
Chen et al., 2022—Early-Onset Colorectal Cancer Is Associated with a Lower Risk of Metachronous Advanced Neoplasia than Traditional-Onset Colorectal Cancer	✓	Prognosis/surveillance/management
Chen et al., 2025—Prognosis and clinicopathological features of patients with early-onset and late-onset colorectal cancer with second primary malignancies	✓	Prognosis/surveillance/management
Cheng et al., 2021—Analysis of Survival Among Adults With Early-Onset Colorectal Cancer in the National Cancer Database	✓	Prognosis/surveillance/management

de Voer et al., 2016—Identification of Novel Candidate Genes for Early-Onset Colorectal Cancer Susceptibility	✓	Hereditary and genetic susceptibility
Demb et al., 2024—Red Flag Signs and Symptoms for Patients With Early-Onset Colorectal Cancer: A Systematic Review and Meta-Analysis	✓	Symptoms and diagnostic pathways
Farooq et al., 2022—Patterns of healthcare utilization leading to diagnosis of young-onset colorectal cancer (yCRC): population-based case-control study	✓	Symptoms and diagnostic pathways
Fritz et al., 2023—Red-flag signs and symptoms for earlier diagnosis of early-onset colorectal cancer	✓	Symptoms and diagnostic pathways
Giráldez et al., 2012—Susceptibility genetic variants associated with early-onset colorectal cancer	✓	Hereditary and genetic susceptibility
Golubicki et al., 2020—Germline biallelic Mcm8 variants are associated with early-onset Lynch-like syndrome	✓	Hereditary and genetic susceptibility
Iberri Jaime et al., 2025—Early-Onset Colorectal Cancer: A New Clinical Profile in Patients Treated at a Referral Hospital in Mexico City	✓	Clinical/anatomical phenotype
Imperiale et al., 2025—A qualitative study of a new metric for estimating early-onset colorectal cancer risk in male veterans: 'Colon Age'	✓	Mixed risk factors and prediction
Kędzia-Berut et al., 2023—Colorectal Cancer: Is it Still a Disease of the Elderly?	✓	Overview and emerging mechanisms

Kolarich et al., 2018—Rectal cancer patients younger than 50 years lack a survival benefit from NCCN guideline-directed treatment for stage II and III disease	✓	Prognosis/surveillance/management
Kolb et al., 2021—Early-Age Onset Colorectal Neoplasia in Average-Risk Individuals Undergoing Screening Colonoscopy: A Systematic Review and Meta-Analysis	✓	Screening and early detection
Laskar et al., 2024—Genome-wide association studies and Mendelian randomization analyses provide insights into the causes of early-onset colorectal cancer	✓	Hereditary and genetic susceptibility
Lin et al., 2025—Early- vs. late-onset colon cancer: clinicopathological insights and survival outcomes in an East Asian cohort	✓	Prognosis/surveillance/management
Malik et al., 2018—Metachronous colorectal cancer following segmental or extended colectomy in Lynch syndrome: a systematic review and meta-analysis	✓	Hereditary and genetic susceptibility
Monge et al., 2024—WNT and TGF-Beta Pathway Alterations in Early-Onset Colorectal Cancer Among Hispanic/Latino Populations	✓	Molecular and tumor biology
Monge et al., 2025—Molecular Heterogeneity in Early-Onset Colorectal Cancer: Pathway-Specific Insights in High-Risk Populations	✓	Molecular and tumor biology

Mueller et al., 2021—Colorectal cancer of the young displays distinct features of aggressive tumor biology: A single-center cohort study	✓	Molecular and tumor biology
Quach et al., 2012—Clinical, endoscopic and pathogical characteristics of early-onset colorectal cancer in Vietnamese	✓	Clinical/anatomical phenotype
Roos et al., 2019—Effects of Family History on Relative and Absolute Risks for Colorectal Cancer: A Systematic Review and Meta-Analysis	✓	Family history and familial polyps
Salem et al., 2020—Molecular Analyses of Left- and Right-Sided Tumors in Adolescents and Young Adults with Colorectal Cancer	✓	Molecular and tumor biology
Song et al., 2021—Risk of colorectal cancer in first degree relatives of patients with colorectal polyps: nationwide case-control study in Sweden	✓	Family history and familial polyps
Tashkandi, 2025—The Rise of Early-Onset Colorectal Cancer: Clinical Characteristics and Outcomes in a Young Patient Population	✓	Clinical/anatomical phenotype
Torres et al., 2025—Unraveling Early Onset Disparities and Determinants: An Analysis of Colorectal Cancer Outcomes and Trends in Texas	✓	Socioeconomic/demographic disparities
van Roon et al., 2010—Early onset MSI-H colon cancer with MLH1 promoter methylation, is there a genetic predisposition?	✓	Hereditary and genetic susceptibility

Vande Perre et al., 2025—Germline Sequencing of Familial and Sporadic Early-Onset Colorectal Cancer: A Novel Pattern of Genes	✓	Hereditary and genetic susceptibility
Wang et al., 2022—High risk and early onset of cancer in Chinese patients with Peutz-Jeghers syndrome	✓	Hereditary and genetic susceptibility
Wang et al., 2023—Identification of specific susceptibility loci for the early-onset colorectal cancer	✓	Hereditary and genetic susceptibility
Yildiz et al., 2023—Genetic insights: High germline variant rate in an indigenous African cohort with early-onset colorectal cancer	✓	Hereditary and genetic susceptibility
Yurgelun et al., 2015—Germline TP53 Mutations in Patients With Early-Onset Colorectal Cancer in the Colon Cancer Family Registry	✓	Hereditary and genetic susceptibility
Zaki et al., 2023—Racial and Ethnic Disparities in Early-Onset Colorectal Cancer Survival	✓	Socioeconomic/demographic disparities
